# Fatty acid profile and safety aspects of the edible oil prepared by artisans' at small‐scale agricultural companies

**DOI:** 10.1002/fsn3.2495

**Published:** 2021-08-08

**Authors:** Elena Bartkiene, Vadims Bartkevics, Zane Berzina, Jolita Klementaviciute, Sonata Sidlauskiene, Ausra Isariene, Vaida Zeimiene, Vita Lele, Erika Mozuriene

**Affiliations:** ^1^ Institute of Animal Rearing Technologies Lithuanian University of Health Sciences Kaunas Lithuania; ^2^ Department of Food Safety and Quality Lithuanian University of Health Sciences Kaunas Lithuania; ^3^ Institute of Food Safety Animal Health and Environment BIOR Riga Latvia; ^4^ State Food and Veterinary Service Vilnius Lithuania; ^5^ National Food and Veterinary Risk Assessment Institute Vilnius Lithuania

**Keywords:** edible oil, fatty acid profile, mycotoxins, polycyclic aromatic hydrocarbons

## Abstract

The aim of this study was to analyze the fatty acid (FA) profiles and mycotoxin and polycyclic aromatic hydrocarbon (PAH) concentrations in sea buckthorn (SB1, SB2), flaxseed (FL3, FL4, FL5), hempseed (HE6, HE7, HE8), camelina (CA9, CA10), and mustard (MU11) edible oils, prepared by artisans’ by artisanal at small‐scale agricultural companies in Lithuania. The dominant FAs were palmitic and oleic acids in SB; palmitic, stearic, oleic, linoleic, and α‐linolenic acids in FL; palmitic, stearic, oleic, linoleic, and α‐linolenic acids in HE; palmitic, oleic, linoleic, α‐linolenic, eicosenoic, and erucic acids in CA; and oleic, linoleic, α‐linolenic, eicosenoic, and erucic acids in MU. In SB2 oil samples, T‐2 toxin and zearalenone concentrations higher than 1.0 µg/kg were found (1.7 and 3.0 µg/kg, respectively). In sample FL4, an ochratoxin A concentration higher than 1.0 µg/kg was established (1.2 µg/kg); also, in HE8 samples, 2.0 µg/kg of zearalenone was found. None of the tested edible oils exceeded the limits for PAH concentration. Finally, because of the special place of edible oils in the human diet, not only should their contamination with mycotoxins and PAHs be controlled but also their FA profile, as an important safety characteristic, must be taken into consideration to ensure higher safety standards.

## Graphical abstract

Edible oils, because of their special place in the human diet, should be controlled not only for contamination with mycotoxins and PAHs but also to ensure higher safety standards. Their FA profiles, as an important safety characteristic, must be taken into consideration

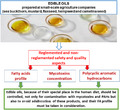

## 1 INTRODUCTION

Nowadays, the popularity of plant‐based edible oils is on the rise; they are gaining the interest of consumers because of their functional and health‐promoting properties (Vasseghian et al., 2020). Moreover, consumers are increasingly choosing nontraditional oils in search of new food sensory properties and greater functionality. For this reason, edible oils prepared from sea buckthorn, mustard, flaxseed, hempseed, and camelina seeds at small‐scale agriculture companies are gaining popularity.

Oil prepared from sea buckthorn has a high content of palmitoleic acid; however, oils prepared from seeds and pulp have a different fatty acid (FA) profile (Ciesarová et al., 2020). Sea buckthorn seeds contain 12.5% oil, and the whole fruits, on average, 10% (Zielińska & Nowak, 2017). In addition, sea buckthorn is rich in tocopherols and other bioactive compounds (Olas, 2018; Tudor et al., 2020).

Mustard seed oil is rich in antioxidants and essential oils, the dominant FAs being oleic, linoleic, and linolenic acids, as well as erucic acid, the last one undesirable as a food component (Mejia‐Garibay et al., 2015, 1990; Peng et al., 2014).

Flaxseed is a very important plant worldwide, and its seeds are considered a “superfood,” a safe source of vitamins, minerals, and bioactive cyclic peptides, as well as various lipids (e.g., polyunsaturated fatty acids (PUFAs), omega‐3, and omega‐6 fats), lignans, and dietary fiber (Bekhit et al., 2018; Goyal et al., 2014; Shim et al., 2014).

Hempseed is rich in vitamins (A, C, and E), micro‐ and macroelements (magnesium, phosphorus, potassium), β‐carotene, protein (on average 23%), soluble fiber (on average 12%), and oil (on average 30%) (Baeck et al., 2019).

Camelina seeds are characterized as a rich source of the n‐3 FA α‐linolenic acid; they are used as a component in the production of foods and supplements with added value and functional properties (Berti et al., 2016; Ibrahim, 2015). In addition to the high content of oleic acid, camelina oil is also rich in tea polyphenols, tocopherols, and phytosterols, which can serve as natural antioxidants and could be used for cardiovascular protection and immunity enhancement (Shen et al., 2021).

The World Health Organization (WHO) reports cardiovascular diseases as the primary cause of death in the world (World Health Organisation, 2021). The evidence on the health effects of total intake of PUFAs, which is the combination of omega‐3 and omega‐6 fats, is equivocal. As cardiovascular diseases are important determinants of health, that particularly burden the poorest people (World Health Organisation, 2021), we need to understand the role of PUFAs to provide the best advice for individuals and populations about how to eat to reduce the risk of ill health. This assessment of the health effects of total PUFA intake is needed alongside an updated assessment of the effects of omega‐3 and omega‐6 fats (Abdelhamid et al., 2018; Hooper et al., 2018).

Recent studies of science and practice confirm the effectiveness of using PUFAs for the prevention and treatment of various diseases, such as lowering of blood pressure; reducing thrombotic tendency; anti‐inflammatory and antiarrhythmic effects; improving vascular endothelial function; increasing plaque stability (through increased plaque calcification); and improving insulin sensitivity (Calder, 2012; Ohwada et al., 2016).

Despite the good safety profile and desirable health aspects of PUFAs (Moloudizargari et al., 2018), including edible oils, they can be contaminated with toxic molds and their metabolites ( Bhat & Reddy, 2017) and *trans* fatty acids (TFAs) (Chen & Liu, 2020). The physiological mechanisms of mycotoxin action in mammalian bodies are very toxic, for example, aflatoxins destabilize protein synthesis and ochratoxins inhibit metabolism involving phenylalanine and zearalenone, leading to estrogenic and teratogenic effects (Sun et al., 2014; Thompson & Raizada, 2018). It is very important to point out that the synergistic activity of mycotoxins leads to multiple, sometimes cumulative, toxic effects; for this reason, the presence of mycotoxins in foodstuffs raises the risk of associated public health concerns (Alassane‐Kpembi et al., 2017). Mycotoxicosis is characterized by an accumulation of the above‐mentioned toxins in body organs, tissues, and the central nervous system (Gherbawy et al., 2012). Low concentrations of aflatoxin can lead to long‐term effects; the most common effect of the majority of mycotoxins is cancerogenic, as DNA replication is influenced by some mycotoxins, and incompatible effects appear. Aflatoxin is involved in immunosuppression and mutagenic, teratogenic, and carcinogenic actions (Fan et al., 2013). The International Agency for Research on Cancer (IARC) indicates that aflatoxin B1 is a Group 1 agent (carcinogen), and ochratoxin belongs to Group 2B (probable carcinogen) (Fashandi et al., 2018; Ostry et al., 2017). The technological steps applied to the refining and extraction of edible oils vary according to the type of edible oil and refining technology. Some have an influence on the mycotoxin concentrations in edible oils and others do not; however, reports of a high occurrence of mycotoxin contamination in edible oils worldwide have been published (Bordin et al., 2014; Cavaliere et al., 2007; Karunarathna et al., 2019; Shephard, 2018). It should be mentioned that nowadays, many consumers select edible oils from nontraditional plants; moreover, products prepared at small‐scale agricultural companies are associated with the characteristics “natural,” “ecological”, and “‘healthier” (sometimes proven, sometimes not). However, in such types of edible oil, as well as the stock from which they are prepared, mycotoxin contamination is not controlled. For this reason, it is very important to know about the challenges in the small‐scale edible oil industry, especially because most of the technological steps included in high‐capacity edible oil technology are not used on a small scale.

According to the European Food Safety Authority (EFSA), TFAs may originate from various sources, including the bacterial conversion of unsaturated FAs in the rumen of ruminants, industrial hydrogenation (used to produce semi‐liquid and solid fats, can be used to produce margarine, shortening, biscuits, etc.), deodorization of unsaturated vegetable oils (or occasionally fish oils) with a high PUFA content (a necessary step of refining), and heating and frying oil at excessively high temperatures (>220°C). TFAs do not play a positive role in any vital functions. On the contrary, the intake of TFAs may harm human health. Evidence suggests that ruminant‐derived TFAs have similar adverse effects on blood lipids and lipoproteins to TFAs from industrial sources. Sufficient evidence is still needed to reveal whether a difference exists between equivalent amounts of ruminant and industrially produced TFAs in terms of the risk of coronary heart disease (EFSA, 2021).

Another challenge related to the safety of edible oils is contamination with polycyclic aromatic hydrocarbons (PAHs). Although the safety of foodis strictly controlled throughout the world (Ji et al., 2020), edible oil is one of the major sources of PAH contamination, due to the hydrophobic characteristics of PAHs (Barranco et al., 2004; Sannino, 2016). PAHs are organic contaminants released through incomplete combustion (Sun et al., 2020) or pyrolysis of organic materials (Drabova et al., 2013). They contain more than one fused aromatic ring (Tfouni et al., 2014), and their toxicity depends on the number of rings: the higher the number of rings, the more toxic and stable the PAH (Li et al., 2003). The 16 most toxic PAHs are indicated as environmental priority pollutants (Zelinkova & Wenzl, 2015), and benzo[a]pyrene (BaP) is indicated as one of the most toxic PAHs (IARC, 2021). It has been suggested that BaP is not an appropriate indicator of the PAH content in food, and four PAHs (BaA—benz[a]anthracene; Chr—chrysene; BbF—benzo[b]fluoranthene; BaP) have been defined which better indicate food contamination with PAHs (EU, 2011). To date, the maximum tolerable value for PAHs in edible oils is 10 µg/kg for the sum of BaA, BaP, Chr, and BbF, and 2 µg/kg for BaP. Finally, data on the PAH concentrations in edible oils produced from sea buckthorn, mustard, flaxseed, hempseed, and camelina seeds at small industrial scale are scarce. For this reason, PAH evaluation in such types of product can lead to solutions of how to increase the safety and quality of the products and to improve their technologies, as well as to give recommendations on edible oil consumption and improve public health.

The aim of this study was to analyze the FA profile, and mycotoxin and PAH concentrations in sea buckthorn, mustard, flaxseed, hempseed, and camelina seed edible oils, prepared at small‐scale companies in Lithuania.

## 2 MATERIALS AND METHODS

### 2.1 Samples of edible oils used for analysis

In total, 11 samples of edible oil were analyzed (Table 1). All of the tested edible oil samples were prepared by artisans' at small‐scale agricultural companies in Lithuania. The oilseeds and sea buckthorn used for oil preparation were also of local (Lithuanian) origin. Usually, this type of company prepares edible oil by cold pressing, without additional extraction with solvents. For this reason, most consumers describe this edible oil as healthier, compared with edible oil obtained by solvent extraction.

**TABLE 1:** Information about oil samples

Sample No.	Type of oil	Code of samples	Country of origin
1	Sea buckthorn	SB1	Lithuania
2		SB2	
3	Flaxseed	FL3	
4		FL4	
5		FL5	
6	Hemp seed	HE6	
7		HE7	
8		HE8	
9	Camelina seeds	CA9	
10		CA10	
11	Mustard seed	MU11	

### 2.2 Fatty acid profile analysis

The FA composition of edible oils was determined using gas chromatography‐flame ionization detection (GC‐FID; Agilent 6890N Gas Chromatograph, Agilent Technologies). Methyl esters of FAs were dissolved in cyclohexane (100 mg in 4 ml), prepared by transmethylation using 8 ml of 1.5% sulfuric acid in methanol, and kept at 60°C for 12 hr. Samples were cooled, shaken for 30 s, centrifuged for 10 min at 3,000 relative centrifugal force at 17°C, and injected (100 µl of the upper part of supernatant, diluted first in cyclohexane 1:9, respectively) into a BPX90 capillary column (60 m × 0.32 mm ID × 0.25 µm film thickness) (SGE, USA). The following parameters were used: flame ionization detector: 280°C; H_2_ flow: 40 ml/min; airflow: 450 ml/min; helium (carrier gas) flow: 1 ml/min; injector: 250°C (split 1:10); oven temperature 50°C (2 min), 4°C min^−1^ to 245°C, and 245°C for 15 min. The identification of FAs was carried out by their retention times and expressed as a percentage of the total peak area of all the FAs in the edible oil sample.

### 2.3 Analysis of mycotoxins in edible oil samples

Deoxynivalenol (DON, 98.3%), aflatoxin B1 (AFB1, 99%), HT‐2 toxin (HT‐2, 99%), T‐2 toxin (T‐2, 99%), zearalenone (ZEN, 99.66%), ochratoxin A (OTA, 99%), and fumonisins B1 and B2 (FB1, 98%; FB2, 97.5%) were acquired from Romer Labs (Tulln). Standard stock solutions of all mycotoxins were prepared in acetonitrile. The spiking solutions and calibration standards were prepared by serial dilution of stock solutions and were stored in UV‐protected glassware at 4°C. The samples (2.50 ± 0.01 g) were accurately weighed in 50‐ml PP tubes. The quality control (blank) samples were supplemented with mycotoxin standard solutions at the appropriate spiking levels. Then, acetonitrile (10 ml) was gradually added to the tubes, and extraction was started by mixing for 5 min on a mechanical shaker. The obtained mixtures were centrifuged (1,313 × *g*, 5 min), and the supernatants were transferred to 15‐ml centrifuge tubes and stored for 15 min at −80°C in a Heto PowerDry^®^ freeze dryer (Thermo Fisher Scientific). After removal, the extracts were immediately centrifuged (2,626 × *g*, 5 min) at 10°C. For each sample, replicate volumes (500 µl) were transferred to 10‐mL glass tubes, whereas the remaining extracts (5 ml) were transferred to QuEChERS dSPE centrifuge tubes for clean‐up. The tubes were shaken for 5 min and centrifuged (2,626 × *g*, 5 min) at room temperature to obtain purified extracts. The initial fractions (500 µl) and the purified extracts (3.5 ml) were pooled and evaporated to dryness at 50°C under a gentle nitrogen stream. The dry residues were reconstructed in 200 µl of injection solution and transferred into the autosampler for analysis. Moldy samples were filtered through centrifuge filters (3,900 × *g*, 10 min) before analysis. High‐performance liquid chromatography/tandem mass spectrometry (HPLC‐MS/MS) analysis was performed on an UltiMate 3,000 (Thermo Fisher Scientific) HPLC system coupled to a TSQ Quantiva MS/MS detector (Thermo Fisher Scientific). Chromatographic separation was performed on a reversed‐phase analytical column (Kinetex C18, 1.7 µm, 100 Å, 50 × 3.00 mm; Phenomenex) at a 0.35 ml/min flow rate. A ternary gradient elution was carried out using 0.1% formic acid in water (eluent A), 0.1% formic acid in methanol (eluent B), and 0.1% formic acid in acetonitrile (eluent C) according to the following gradient program: 0–1.5 min: 0% B and 30% C; 2.0–2.7 min: 15% B and 35% C; 5.5–6.5 min: 40% B and 58% C; 8.0 min: 5% B and 93% C; 8.5–9.5 min: 0% B and 10% C; 10.0 min: 0% B and 30% C. The autosampler was maintained at 4°C, and the column temperature was 40°C. The sample injection volume was 15 µl. Ion monitoring was conducted in both positive and negative ion modes, and the mass analysis was performed in selective reaction monitoring (SRM) mode. The following instrumental settings were used: spray voltage 3.5 kV (positive ion mode); 2.5 kV (negative ion mode); vaporizer temperature 350°C; ion transfer temperature 300°C; sheath gas 55 arbitrary units (arb); auxiliary gas 25 arb; and sweep gas 5 arb. Data processing was performed with Xcalibur™ software (Thermo Fisher Scientific).

### 2.4 Determination of polycyclic aromatic hydrocarbons in edible oil samples

The solvents employed were cyclohexane, hexane, dichloromethane, and ethyl acetate, all of which were of pesticide purity grade. Other reagents and materials used were anhydrous sodium sulfate and 6 ml (500 mg) Phenomenex Strata SI‐1 Silica solid phase extraction (SPE) tubes. All the aforementioned solvents, reagents, and materials were commercially purchased from Sigma‐Aldrich (Steinheim, Germany), Supelco, and Merck. The mixture of four PAH standards: BaA, BbF, BaP,and Chr, and deuterated standards BaP‐d12, BbF‐d12, Chr‐d12, and BaA‐d12 were purchased from Dr. Ehrenstrofer. The standard mix of PAHs consisted of a 50 mg/L solution in acetonitrile, and the concentration of deuterated BaP‐d12, BbF‐d12, Chr‐d12, BaA‐d12 dissolved in cyclohexane was 1,000 ng μl^−1^. The mixtures were stored at 4°C. The PAH analysis was carried out according to Rozentale et al. (2015) using a Thermo Scientific TSQ Quantum XLS Ultra GCeMS/MS system equipped with a DB‐17 capillary column (30 m long × 0.25 mm i.d. × 0.25 μm film thickness) and operating in splitless mode. The operating conditions were as follows: Helium gas was used as the carrier gas at a constant flow of 1.2 ml/min; inlet temperature 260°C; MS transfer line temperature 280°C; source temperature 250°C. The oven temperature was set initially at 80°C (2 min hold), increased to 265°C at 15°C min^−1^. At 265°C, the temperature was increased at a rate of 5°C min^−1^–290°C and then to 320°C at a rate of 20°C min^−1^ (20 min hold). The total run time was 45.8 min. The injection volume was 1 μl. The data were acquired by operating the MS in SRM mode.

### 2.5 Statistical analysis

In order to evaluate the influence of the type of edible oil on the FA profile, and mycotoxin and PAH concentrations, data were analyzed by one‐way ANOVA (statistical program R 3.2.1). The results were recognized as statistically significant at *p* ≤ .05.

## 3 RESULTS

### 3.1 Fatty acid profiles of sea buckthorn, flaxseed, hempseed, and camelina seed oils

The FA profiles of sea buckthorn (SB1 and SB2), flaxseed (FL3, FL4, and FL5), hempseed (HE6, HE7, and HE8), camelina seed (CA9 and CA10), and mustard (MU11) edible oils are shown in Table 2. In a comparison of samples SB1 and SB2, a significantly higher content of palmitoleic, heptadecanoic, ginkgolic, oleic, gondoic, arachidic, and lignoceric acids was found in SB2 samples (on average, 1.3, 0.2, 3.7, 3.5, 1.9, 1.8, and 1.9 times higher). In contrast to the above‐mentioned FAs, in sample SB2, a significantly lower content of stearic, linoleic, and α‐linolenic acids was established than in sample SB1 (on average, 140.7, 4.7, and 2.7 times lower). In sample SB1, saturated fatty acid (SFA), PUFA, omega‐3, and omega‐6 FA concentrations were 22.2%, 78.5%, 62.3%, and 78.7% higher, respectively, than in SB2. However, in sample SB2, the monounsaturated fatty acid (MUFA) and omega‐9 FA content were higher (on average, by 70.5% and 71.2%, respectively) than that in SB1. The dominant FAs in SB samples were palmitic, oleic, and C18:1 omega‐6 (on average, in sample SB1 10.71%, 20.41%, and 60.97% of the total fat content, respectively, and in sample SB2 10.51%, 71.85%, and 12.98% of the total fat content, respectively).

**TABLE 2:** Fatty acid profile of the sea buckthorn, flax, hemp, and camelina seed oils

Oil samples											
Fatty acids	SB1	SB2	FL3	FL4	FL5	HE6	HE7	HE8	CA9	CA10	MU11
	Fatty acids concentration, % from total fat content										
C14:0	0.14 ± 0.02 b,D	0.10 ± 0.01 a,C	0.05 ± 0.00 a,A	0.05 ± 0.00 a,A	0.05 ± 0.01 a,A	0.04 ± 0.01 a,A	0.04 ± 0.01 a,A	0.05 ± 0.01 a,A	0.06 ± 0.01 a,A,B	0.06 ± 0.0 a,A,B	0.07 ± 0.01 B
C16:0	10.71 ± 0.90 a,E	10.51 ± 0.54 a,E	6.84 ± 0.20 a,C	6.94 ± 0.20 a,C	6.83 ± 0.19 a,C	7.22 ± 0.24 a,C,D	8.20 ± 0.25 b,D	7.59 ± 0.30 a,C,D	5.73 ± 0.18 a,B	5.90 ± 0.08 a,B	2.99 ± 0.09 A
C16:1 ω7	0.86 ± 0.10 a,E	1.15 ± 0.06 b,D	0.10 ± 0.01 b,A	0.09 ± 0.01 a,A	0.08 ± 0.01 a,A	0.16 ± 0.01 a,C	0.14 ± 0.06 a,C	0.17 ± 0.01 a,C	0.13 ± 0.01 a,B,C	0.11 ± 0.00 a,B	0.18 ± 0.01 C
C17:0	0.06 ± 0.01 a,C	0.09 ± 0.03 b,D	0.07 ± 0.00 a,D	0.07 ± 0.01 a,D	0.07 ± 0.01 a,D	0.06 ± 0.00 a,C	0.06 ± 0.00 a,C	0.06 ± 0.00 a,C	0.05 ± 0.00 a,B	0.04 ± 0.00 a,B	0.02 ± 0.00 A
C17:1	0.03 ± 0.01 a,B	0.11 ± 0.02 b,C	0.04 ± 0.00 a,B	0.04 ± 0.01 a,B	0.04 ± 0.00 a,B	0.03 ± 0.01 a,B	0.03 ± 0.00 a,B	0.03 ± 0.00 a,B	0.02 ± 0.00 a,A	0.02 ± 0.00 a,A	0.06 ± 0.01 BC
C18:0	4.22 ± 0.33b,E,F	0.03 ± 0.02 a,A	7.14 ± 0.31 b,G	5.39 ± 0.16 a,F	5.41 ± 0.30 a,F	2.99 ± 0.20 a,D	3.66 ± 0.26 b,E	2.89 ± 0.23 a,D	2.93 ± 0.22 b,D	2.51 ± 0.07 a,C	1.08 ± 0.06 B
C18:1 ω9	20.41 ± 0.84 a,F	71.85 ± 0.71 b,G	15.35 ± 0.75 c,E	12.24 ± 0.14 b,D,E	11.27 ± 0.14 a,D	8.08 ± 0.15 b,A,B	8.70 ± 0.18 c,B	7.62 ± 0.25 a,A	10.71 ± 0.05 b,C,D	9.09 ± 0.02 a,C	14.58 ± 0.35 E
C18:2 ω6	nd	nd	nd	nd	nd	3.58 ± 0.16 b,C	5.45 ± 0.22 c,D	3.06 ± 0.18 a,B	nd	nd	0.03 ± 0.00 A
C18:2 ω6	60.97 ± 0.55 b,F	12.98 ± 0.94 a,B	20.19 ± 0.49 b,D	18.10 ± 0.17 a,C	17.81 ± 0.20 a,C	49.63 ± 0.67 a,E	50.07 ± 0.85 a,E	49.61 ± 1.08 a,E	17.48 ± 0.11 a,C	17.09 ± 0.10 a,C	11.00 ± 0.07 A
C18:3 ω3	0.77 ± 0.14 b,B	0.29 ± 0.06 a,A	49.40 ± 0.75 a,G	55.22 ± 0.67 b,H	57.74 ± 0.72 c,I	26.45 ± 0.34 b,E	21.59 ± 0.53 a,D	27.16 ± 0.28 b,E	37.59 ± 0.98 a,F	38.34 ± 0.42 a,F	13.66 ± 0.09 C
C20:0	0.38 ± 0.05 a,B	0.40 ± 0.10 a,B	0.23 ± 0.03b,A,B	0.16 ± 0.02 a,A	0.15 ± 0.01 a,A	0.84 ± 0.08 a,D	1.07 ± 0.10 b,E	0.84 ± 0.09 a,D	1.41 ± 0.07 b,F	1.25 ± 0.06 a,E	0.65 ± 0.06 C
C20:1 ω9	0.36 ± 0.09 a,B	0.70 ± 0.13 b,C	0.33 ± 0.03 b,B	0.33 ± 0.02 b,B	0.15 ± 0.01 a,A	0.46 ± 0.07 a,C	0.51 ± 0.09 a,C	0.49 ± 0.09 a,C	14.95 ± 0.12 a,E	14.82 ± 0.11 a,E	9.49 ± 0.11 D
C20:2 ω6	0.03 ± 0.01 A	nd	0.04 ± 0.00 a,A	0.03 ± 0.00 a,A	0.03 ± 0.00 a,A	0.09 ± 0.01 a,B	0.09 ± 0.01 a,B	0.10 ± 0.01 a,B	2.40 ± 0.17 a,D	2.63 ± 0.05 a,D	0.35 ± 0.06 C
C22:0	0.59 ± 0.08 a,E	1.04 ± 0.12 b,F	0.10 ± 0.01 b,B	0.07 ± 0.01 a,A	0.06 ± 0.00 a,A	0.16 ± 0.01 a,C	0.17 ± 0.01 a,C	0.15 ± 0.01 a,C	0.14 ± 0.01 a,C	0.14 ± 0.01 a,C	0.25 ± 0.02 D
C22:1 ω9	0.16 ± 0.16 a,B	0.15 ± 0.21 a,B	0.03 ± 0.01 a,A	1.18 ± 0.14 c,C	0.22 ± 0.07 b,B	0.06 ± 0.04 a,A	0.07 ± 0.05 a,A	0.07 ± 0.04 a,A	5.63 ± 0.27 a,D	7.20 ± 0.11 b,E	42.66 ± 0.21 F
C24:0	0.31 ± 0.08 a,D	0.60 ± 0.04 b,E	0.09 ± 0.02 a,A	0.09 ± 0.01 a,A	0.08 ± 0.01 a,A	0.14 ± 0.01 a,B	0.14 ± 0.02 a,B	0.12 ± 0.01 a,B	0.18 ± 0.02 a,C	0.17 ± 0.01 a,C	0.26 ± 0.05 D
C24−1 ω9	nd	nd	nd	nd	nd	nd	nd	nd	0.59 ± 0.07 a,A	0.63 ± 0.07 a,A	2.67 ± 0.16 B
SFAs	16.41	12.77	14.51	12.77	12.66	11.45	13.35	11.70	10.50	10.08	5.32
MUFAs	21.82	73.96	15.85	13.88	11.76	8.80	9.45	8.38	32.02	31.87	69.65
PUFAs	61.77	13.27	69.64	73.35	75.58	79.76	77.20	79.93	57.48	58.06	25.03
Omega−3	0.77	0.29	49.40	55.22	57.74	26.45	21.59	27.16	37.59	38.34	13.66
Omega−6	61.00	12.98	20.23	18.13	17.84	53.30	55.61	52.77	19.89	19.71	11.38
Omega−9	20.92	72.71	15.71	13.75	11.64	8.60	9.28	8.17	31.87	31.74	69.40

Note

Data are represented as means (*n* = 5) ± SE.

Abbreviations: CA, camelina seeds oil; FL, flaxseed oil; HE, hemp seed oil; MU, mustard seed oil; MUFAs, monounsaturated fatty acids; PUFAs, polyunsaturated fatty acids; SB, sea buckthorn oil; SFAs, saturated fatty acids.

^a‐c^Mean values within a the same group of samples in the column with different letters are significantly different (*p* ≤ .05).

^A‐F^Mean values within a column with different letters are significantly different (*p* ≤ .05).

Due to the well‐balanced FA profile (unique SFAs palmitoleic acid (PA; 16:1 omega‐7) and omega‐6) and high concentration of carotenoids and vitamins, SB oil is used in the cosmetic industry (Koskovac et al., 2017; Zielińska & Nowak, 2017) and has been shown to exert preventative effects in hypercholesterolemia, diabetes, and liver dysfunction (Solà Marsiñach & Cuenca, 2019). The SB FA profile may differ depending on origin, subspecies, harvesting time, etc. (Kuhkheil et al., 2018; Solà Marsiñach & Cuenca, 2019). It should be mentioned that the FAs determined in sample SB1 in this study were similar to the FA profile of sunflower oil (Sanyal et al., 2018; Wang et al., 2018); moreover, the FAs in sample SB2 were similar to the FA profile of olive oil (Wani et al., 2018). According to these results, it can be stated that samples SB1 and SB2 were not pure sea buckthorn oil, and they were diluted with sunflower and olive oil, respectively. According to Burčová et al. (Burčová et al., 2017), the predominant unsaturated fatty acids (UFAs) in SB seeds are linoleic (37.3%), α‐linolenic (29.9%), and vacceniccids (20.3%). Kuhkheil et al. (Kuhkheil et al., 2018) reported averages of 15.76%–17.01% for palmitic, 6.46%–7.72% for hexadecenoic, 9.68%–15.42% for oleic, and 23.76%–23.82% for linolenic acid in SB seed oil (% of the total fat content). According to Crăciun (Crăciun, 2018), the percentages of palmitic, hexadecenoic, oleic, and linoleic acid in SB oil were 35.01%, 27.7%, 22.52%, and 3.7%, respectively.

In a comparison of samples FL3, FL4, and FL5, the FL3 group showed a significantly higher content of palmitoleic, stearic, oleic, linoleic, arachidic, and arachidic acids; however, in sample FL3, the α‐linolenic and erucic acid content was the lowest in the flaxseed oil sample group. Moreover, the content of SFAs, MUFAs, omega‐6, and omega‐9 FAs was the highest in sample FL3 (on average, 12.3%, 19.1%, 11.1%, and 19.2% higher, respectively). In contrast, a higher content of PUFAs and omega‐3 FAs was found in samples FL4 and FL5 (by 6.5% and 12.5%, respectively, in comparison with FL3). The dominant FAs in flaxseed oil samples were palmitic, stearic, oleic, linoleic, and α‐linolenic acids; their content in FL3, FL4, and FL5 was, on average, 6.87%, 5.98%, 12.95%, 18.70%, and 54.12% of the total fat content, respectively.

Many beneficial effects of FL have been reported (reducing insulin and increasing total antioxidant capacity, anticoagulant, and antihypertensive properties, regulation of lipid metabolism, supporting the central nervous system, improving eyesight, etc.) (Raygan et al., 2019; Sokoła‐Wysoczańska et al., 2018). In addition, FL can be used in anti‐cancer therapy (Buckner et al., 2019). FL is rich in the omega‐3 PUFA α‐linolenic acid (ALA) (Nasirpour‐Tab rizi et al., 2020; Yadav et al., 2021; Zhu et al., 2020). Our results are in accordance with those of Nasirpour‐Tabrizi et al. (Nasirpour‐Tab rizi et al., 2020), who published that the omega‐3 essential FA ALA comprises about 59% of the total FAs of FL.

In a comparison of samples HE6, HE7, and HE8, the highest content of palmitic, stearic, oleic, linoleic, and arachidic acids was found in sample HE7. However, the lowest ALA content was established in HE7. The highest oleic acid content was found in HE7. Sample HE7 showed the highest SFA, MUFA, omega‐6, and omega‐9 concentrations; however, the highest content of PUFAs and omega‐3 was found in sample HE8. The dominant FAs in the hemp seed oil sample group were palmitic, stearic, oleic, linoleic, and ALA, and their content in samples HE6, HE7, and HE8 was, on average, 7.67%, 3.18%, 8.13%, 49.77%, and 25.07% of the total fat content, respectively.

The profile of hempseed oil is very specific as, on average, 79% of its FAs are PUFAs, of which linoleic acid (54%) and ALA (19%) are dominant (Da Porto et al., 2015; Moczkowska et al., 2020; Pratap Singh et al., 2020; Rezvankhah et al., 2019). In this study, the data obtained are in accordance with the above‐mentioned reported data. Hempseed oil, due to its high amount of PUFAs, is used to enrich foods, as well as in the nutraceutical, pharmaceutical, supplement, etc. industries (Rezvankhah et al., 2019). Omega‐3 FAs contribute to a reduction in the risk of cardiovascular diseases and pro‐inflammatory cytokines (Moura‐Assis et al., 2018), also showing beneficial effects on gene expression related to insulin metabolism, lipids and inflammation, glycemic control, and oxidative stress (Jamilian et al., 2020).

The main differences found between the FA profiles of camelina seed oil samples CA9 and CA10 were in the stearic, oleic, arachidic, and erucic acid content. The highest content of stearic, oleic, and arachidic acids was found in CA9 samples, and the highest erucic acid content was established in sample CA10. Sample CA9 showed a higher content of SFAs, MUFAs, omega‐6, and omega‐9 FAs; however, a higher content of PUFAs and omega‐3 FAs was found in sample CA10. The dominant FAs in camelina seed oil samples were palmitic, oleic, linoleic, ALA, gondoic, and erucic acids, and their content in samples CA9 and CA10 was, on average, 5.82%, 9.9%, 17.29%, 37.97%, 14.89%, and 6.42% of the total fat content, respectively.

*Camelina sativa* oil is rich in vitamins, UFAs, phytosterols, and polyphenols (Kurasiak‐Popowska & Stuper‐Szablewska, 2020). In accordance with our results and those of other authors, the dominant FAs in camelina oil are UFAs, MUFAs, and mostly PUFAs (>55%), SFAs ranging from 9.1% to 10.8% (Popa, et al., 2021). The most frequent FAs identified in camelina oil are linolenic (on average, 35%), linoleic (on average, 20.5%), oleic (on average, 16%), and eicosenoic (on average, 17%) (Popa, et al., 2021). Ratusz et al. (Ratusz et al., 2018) analyzed 29 cold‐pressed camelina oils and determined a highly optimal n‐3 PUFA to n‐6 PUFA ratio (1.79–2.17). The major antinutritional compounds in camelina are glucosinolates, tannins, and erucic acid (Singh et al., 2021). Kurasiak‐Popowska and Stuper‐Szablewska ( Kurasiak‐Popowska & Stuper‐Szablewska, 2020) found that the average content of erucic acid is 3.43% in the spring genotypes and 0.1% in the winter genotypes. However, in our study in the tested CA samples, the erucic acid content was, on average, 6.42%.

The dominant FAs s mustard seed oil (MU11) were oleic, linoleic, ALA, gondoic, and erucic acids (14.58%, 11.00%, 13.66%, 9.49%, and 42.66% of total fat content, respectively). The SFA content in MU11 was, on average, 5.32% of the total fat content, and the predominant FAs in MU11 were MUFAs (69.65% of the total fat content). The PUFA content in MU11 was, on average, 2.9 times lower than the MUFA content. Comparing FA series, the most abundant in MU11 was omega‐9 (69.40% of total fat content), and the omega‐3 and 6 content in MU11 was, on average, 5.1 and 6.1 times lower, respectively.

Our results are in agreement with Stamenković et al. (Stamenković et al., 2018) and Mitrović et al. (Mitrović et al., 2020), who reported that the main FAs in mustard seed oil are UFAs (oleic, eicosenoic, erucic, linoleic, and linolenic). The main specific characteristic of mustard seed oil is its high content of erucic acid, which can range from 32.81% to 60.29% (Mitrović et al., 2020). Erucic acid is a long‐chain FA, classified as a natural toxin due to its detrimental effects on heart muscle function (Vetter et al., 2020) and lipid degeneration of the heart (Krist, 2020). Oxidation of mitochondrial FAs plays a key role in liver lipid metabolism; therefore, it is possible that hepatic metabolism of erucic acid might decrease mitochondrial FA oxidation (Chen et al., 2020). One of the main sources of erucic acid in the human diet is oil prepared from Brassicaceae plants, for example, mustard (Vetter et al., 2020). According to EFSA recommendations, the tolerable daily intake of erucic acid is 7 mg/kg of body weight (EFSA, 2016).

Finally, it can be stated that the FA profile of an edible oil is a very important characteristic, which shows not only a functional aspect but also a safety aspect. The data on FAs in edible oils should be disseminated to a wide audience and, if some of the oils are not recommended for daily consumption as food ingredients, perhaps they could be used in other industries, for example, for cosmetology, etc.

### 3.2 Mycotoxin contamination in tested edible oil samples

The major mycotoxins in food are aflatoxins, OTA, ZEN, fumonisins, and trichothecenes (Vasseghian et al., 2020). Mycotoxin contamination of the tested edible oil samples is shown in Table 3. In SB2, T‐2 and ZEN concentrations higher than 1.0 µg/kg were found (1.7 and 3.0 µg/kg, respectively). As the awareness and understanding of ZEN exposure‐associated risks have increased, the European Commission (EC) has established and enforced a maximum 400 µg/kg ZEN level in refined corn oil, and the tolerable daily intake (TDI) of ZEN has been set at 0.25 µg/kg b.w. based on collected toxicity assessment and exposure data (EC, 2021). ZEN shows distinct lipophilic properties, in contrast to the high water solubility of trichothecenes (Lacko‐Bartošová et al., 2017), and this characteristic can facilitate absorption through the gut. One of the most toxic mycotoxins is T‐2, which is a metabolite of *F. acuminatum* and *F. equiseti*, mainly found in cold climate regions (Kang et al., 2020; Ling et al., 2020). T‐2 is harmful to mammals, and its lipophilic characteristics imply that it is easily absorbed through the gut, skin, and pulmonary mucosa (Sun et al., 2020). Based on these toxic effects, the Panel on Contaminants in the Food Chain (CONTAM Panel) of EFSA set the TDI for the sum of T‐2 and HT‐2 at 100 ng/kg body weight (EU, 2013). In sample FL4, an OTA concentration higher than 1.0 µg/kg was established (1.2 µg/kg). The limits for OTA range from 0 to 50 µg/kg in food (Mazumder & Sasmal, 2001). Ochratoxin is associated with immunotoxic, teratogenic, ascertained nephrotoxic, and carcinogenic effects (Meucci et al., 2021). In oil sample HE8, 2.0 µg/kg of ZEN was determined. ZEN is a metabolite of *Fusarium* species; its estrogenic activity, hepatotoxicity, teratogenicity, genotoxicity, carcinogenicity, hematotoxicity, and immunotoxicity to mammals are well known (Alshannaq & Yu, 2017; Gallo et al., 2015; Häggblom & Nordkvist, 2015; Kowalska et al., 2016).

**TABLE 3:** Mycotoxins contamination (µg/kg) in tested oil samples

Oil samples	*Mycotoxins*							
	DON	AFB1	HT−2	T−2	FB1	FB2	OTA	ZEA
	µg/kg							
SB1	<1	<1	<5	<1	<1	<1	<1	<1
SB2	<1	<1	<5	1.7 ± 0.1	<1	<1	<1	3.0 ± 0.2
FL3	<1	<1	<5	<1	<1	<1	<1	<1
FL4	<1	<1	<5	<1	<1	<1	1.2 ± 0.1	<1
FL5	<1	<1	<5	<1	<1	<1	<1	<1
HE6	<1	<1	<5	<1	<1	<1	<1	<1
HE7	<1	<1	<5	<1	<1	<1	<1	<1
HE8	<1	<1	<5	<1	<1	<1	<1	2.0 ± 0.1
CA9	<1	<1	<5	<1	<1	<1	<1	<1
CA10	<1	<1	<5	<1	<1	<1	<1	<1
MU11	<1	<1	<5	<1	<1	<1	<1	<1

Note

Data are represented as means (*n* = 5) ± SE.

Abbreviations: A; AFB1, Aflatoxin B1; CA, camelina seeds oil; DON, Deoxynivalenol; FB1, Fumonisin B1; FB2, Fumonisin B2; FL, flaxseed oil; HE, hemp seed oil; HT‐2, HT‐2 toxin; MU, mustard seed oil; OTA, Ochratoxin; SB, sea buckthorn oil; T‐2, T‐2 Toxin; ZEA, Zearalenone.

Mycotoxins are thermostable toxins, resistant to high pressure, transportation conditions, etc. (Amirahmadi et al., 2017; Heshmati et al., 2019). The formation of mycotoxins depends not only on the fungal strain but also on environmental conditions, and the reasons for the low concentration of these fungal metabolites in the tested edible oils may be associated with low fungal contamination of the raw material. There is a set maximum concentration for mycotoxin contamination in some foods (Li et al., 2016; Nabizadeh et al., 2018). However, mycotoxin concentrations in sea buckthorn, flaxseed, hempseed, camelina, and mustard seed oils are not regulated. This study, for the first time, presents mycotoxin concentrations in the above‐mentioned oils. Moreover, it should be mentioned that the tested samples were obtained from small local producers, which do not use many technological steps in the process of oil purification. Usually, local producers offer consumers nonrefined products, which are considered healthier and safer options. However, there have been publications about ZEN and trichothecene contamination in both nonrefined and refined oils from soybean, sunflower, and corn germ (Schollenberger et al., 2008). Also, refining cannot protect against *Fusarium* mycotoxin contamination of edible oils (Kamimura et al., 1986).

Assessment of mycotoxin contamination usually focuses on the main food products and the main mycotoxins for which regulatory limits have been set to protect human health (Fontaine et al., 2015). Therefore, further research is needed, as the results of the present study suggest minor contamination of some of the tested edible oils with ZEN, T‐2, and OTA. However, contamination of raw material with fungi is usually due to climatic conditions and many other agricultural factors. For this reason, not only oils but also seeds, as the raw material for oil preparation, must be controlled. Finally, considering that the popularity of edible oils prepared from various nontraditional raw materials is growing, and that such types of product are associated with a healthy lifestyle, it is very important to ensure their safety in terms of mycotoxin contamination.

### 3.3 Polycyclic aromatic hydrocarbon contamination of tested oil samples

The PAH contamination in the tested edible oils is shown in Table 4. In a comparison of the BaA concentration in edible oil samples, the highest concentration was found in two out of three analyzed hemp seed oil samples (HE6 and HE7) and in one out of two analyzed camelina seed oil samples (CA10) (1.41, 2.29 and 1.94 µg/kg, respectively). In samples SB1, FL3, FL4, and HE8, the concentration of BaA was, on average, 0.45 µg/kg. On average, in comparison with the above‐mentioned edible oil samples, the BaA concentrations in samples FL5 (0.76 µg/kg) and SB2 (0.80 µg/kg) were two times higher. A BaA concentration lower than 0.15 µg/kg was found in CA9 and MU11 (0.09 and 0.12 µg/kg, respectively).

**TABLE 4:** Polycyclic aromatic hydrocarbons contamination (µg/kg) in tested oil samples

Oil samples	Polycyclic aromatic hydrocarbons				
	BaA	Chr	BbF	BaP	∑PAHs
	µg/kg				
SB1	0.49 ± 0.10 a,B	1.70 ± 0.34 a,C	0.22 ± 0.04 a,B	0.30 ± 0.06 a,B	2.71
SB2	0.80 ± 0.16 b,C	1.97 ± 0.39 a,C	0.48 ± 0.10 b,C	0.23 ± 0.05 a,B	3.48
FL3	0.48 ± 0.10 a,B	1.03 ± 0.21 a,B	0.51 ± 0.10 a,C	0.29 ± 0.06 b,B	2.31
FL4	0.40 ± 0.08 a,B	0.82 ± 0.16 a,B	0.39 ± 0.08 a,C	0.14 ± 0.03 a,A	1.75
FL5	0.76 ± 0.15 b,C	0.80 ± 0.16 a,B	0.36 ± 0.07 a,C	0.35 ± 0.07 b,B	2.27
HE6	1.41 ± 0.28 b,D	2.14 ± 0.43 b,D	2.08 ± 0.42 a,D	0.81 ± 0.16 a,C	6.44
HE7	2.29 ± 0.46 c,E	3.19 ± 0.64 b,D	2.29 ± 0.46 a,D	1.68 ± 0.34 b,E	9.45
HE8	0.43 ± 0.09 a,B	0.92 ± 0.18 a,B	0.39 ± 0.08 a,C	0.58 ± 0.12 a,C	2.32
CA9	0.09 ± 0.02 a,A	0.21 ± 0.04 a,A	0.05 ± 0.01 a,A	0.21 ± 0.04 a,A	0.56
CA10	1.94 ± 0.39 b,D,E	4.40 ± 0.88 b,D	1.89 ± 0.38 b,D	1.00 ± 0.20 bD	9.23
MU11	0.12 ± 0.02 A	0.26 ± 0.05 A	0.04 ± 0.01 A	0.32 ± 0.06 B	0.74

Note

Data are represented as means (*n* = 3) ± SE.

Abbreviations: ∑PAHs, sum of polycyclic aromatic hydrocarbons; BaA ‐ benz[a]anthracene; BbF, benzo‐[b]fluoranthene; BaP, benzo[a]pyrene; CA, camelina seeds oil; Chr, chrysene; FL, flaxseed oil; HE, hemp seed oil; MU, mustard seed oil; PAHs, polycyclic aromatic hydrocarbons; SB, sea buckthorn oil.

^a‐c^Mean values within a the same group of samples in the column with different letters are significantly different (*p* ≤ .05).

^A‐F^ – Mean values within a column with different letters are significantly different (*p* ≤.05).

The lowest Chr concentration was established in samples CA9 and MU11 (0.21 and 0.26 µg/kg, respectively). In samples FL3, FL4, FL5, and HE8, the average Chr concentration was 0.89 µg/kg. On average, the Chr concentration found in SB1 and SB2 was 1.9 times higher than in the above‐mentioned samples. In contrast to SB samples, the highest Chr concentration was established in HE6, HE7, and CA10 (on average, 3.2 µg/kg).

The concentration of BbF in the tested edible oils ranged from, on average, 0.05 µg/kg (in CA9 and MU11 samples) to 2.09 µg/kg (in HE6, HE7, and CA10 samples).

The lowest BaP concentration was found in samples FL4 and CA (on average, 0.18 µg/kg). HE7 samples showed the highest BaP concentration (1.68 µg/kg), and in samples SB1, SB2, FL3, FL5, and MU11, the average BaP concentration was 0.30 µg/kg. These results are satisfactory for determinations at mg/kg level and comply with the performance criteria for the methods of BaP analysis proposed by the European Union, where the LOD must be lower than 0.3 mg/kg (EC, 2021).

In a comparison of ∑PAHs in the tested edible oil samples, the highest ∑PAH concentration was found in samples HE7 and CA10 (on average, 9.34 µg/kg). The lowest ∑PAHs was shown in samples CA9 and MU11 (0.56 and 0.74 µg/kg, respectively). The results of ANOVA indicated that the separate PAH concentrations and ∑PAHs were significantly influenced by the type of oil (*p* ≤.05) (Table 5). Finally, not one of the tested edible oil samples exceeded the PAH concentration limits, which are for BaP in oil samples ˂2 µg/kg and for ∑PAHs ˂ 10 µg/kg.

**TABLE 5:** Influence of the type of oil on polycyclic aromatic hydrocarbons contamination in tested oil samples

Factor	PAHs	F	*p*
Type of oil	BaA	3.181	.028
	Chr	2.854	.042
	BbF	5.535	.002
	BaP	6.891	.001
	∑PAHs	3.865	.013

Note

Influence of the factor is significant, when *p* ≤ .05.

Abbreviations: ∑PAHs, summa of polycyclic aromatic hydrocarbons; BaA, benz[a]anthracene; BaP, benzo[a]pyrene; BbF, benzo‐[b]fluoranthene; Chr, chrysene; PAHs, polycyclic aromatic hydrocarbons.

European regulation sets limits for some PAHs for the category of oils and fats, that is, 2.0 mg/kg for BaP and 10.0 mg/kg for PAH4 (EC, 2011). PAHs may be generated during stock pretreatment (usually drying), and stock that is already contaminated with PAHs may further spread the contamination to the final product, edible oils (Lee et al., 2020). Edible oils are consumed directly, to improve the organoleptic properties of food or for thermal treatment of food (roasting). PAHs consumed in the diet are easily absorbed through the intestinal tract (Stavric & Klassen, 1994). Usually, contamination of edible oils with PAHs is a consequence of environmental pollution of the raw oilseeds (Drabova et al., 2013; Menichini et al., 2015, 1990), technological processes (contact with direct smoke during the drying process and solvent extraction), or the introduction of nonfood grade mineral oils (Hollosi & Wenzl, 2011). PAHs can be generated during high‐temperature and long‐duration frying (Zhao et al., 2013). In any case, because of the special aspects of edible oils in the human diet, analysis of PAHs in edible oils is necessary (Mohammadi et al., 2020).

## 4 CONCLUSIONS

The dominant FAs were palmitic, oleic, and linoleic acids in SB oil samples; palmitic, stearic, oleic, linoleic, and ALA in FL oil; palmitic, stearic, oleic, linoleic, and ALA in HE; palmitic, oleic, linoleic, ALA, gondoic, and erucic acids in CA; and oleic, linoleic, ALA, gondoic, and erucic acids in MU. According to the results obtained, the FA profile is a very important safety characteristic of an edible oil, and if some of the oils are not recommended for daily consumption as food ingredients, perhaps they could be used in other industries, for example, cosmetology, taking into account their other desirable bioactive compounds. Concentrations of 1.7 µg/kg T‐2 and 3.0 µg/kg ZEN in SB2 oil samples, and 1.2 µg/kg OTA in FL4 and 2.0 µg/kg ZEN in HE8 oil were found. The type of edible oil was a significant factor (*p* ≤.05) for separate PAH concentrations, as well as ∑PAHs; however, none of the tested edible oils exceeded the upper limits for PAH concentrations (for BaP content in oil samples ˂ 2 µg/kg and for ∑PAHs ˂ 10 µg/kg). Finally, because of the special place of edible oils, in the human diet, not only should their contamination with mycotoxins and PAHs be controlled but also their FA profile must be taken into consideration to avoid adulteration of these products.

## CONFLICTS OF INTEREST

The authors declare no conflict of interest.

## AUTHOR CONTRIBUTIONS

**Elena Bartkiene:** Conceptualization (equal); Project administration (equal); Supervision (equal); Writing‐original draft (equal). **Vadims Bartkevics:** Conceptualization (equal); Methodology (equal); Writing‐review & editing (equal). **Zane Berzina:** Formal analysis (equal); Methodology (equal). **Jolita Klementaviciute:** Formal analysis (equal); Writing‐original draft (equal). **Sonata Sidlauskiene:** Formal analysis (equal). **Ausra Isariene:** Writing‐review & editing (equal). **Vaida Zeimiene:** Writing‐review & editing (equal). **Vita Lele:** Writing‐original draft (equal). **Erika Mozuriene:** Visualization (equal); Writing‐original draft (equal).
